# Long-Term Effects of Perinatal Exposure to a Glyphosate-Based Herbicide on Melatonin Levels and Oxidative Brain Damage in Adult Male Rats

**DOI:** 10.3390/antiox12101825

**Published:** 2023-10-03

**Authors:** Daiane Cattani, Paula Pierozan, Ariane Zamoner, Eva Brittebo, Oskar Karlsson

**Affiliations:** 1 Science for Life Laboratory, Department of Environmental Sciences, Stockholm University, 114 18 Stockholm, Sweden; daiane.cattani@aces.su.se (D.C.); paula.pierozan@aces.su.se (P.P.); 2 Department of Pharmaceutical Biosciences, Uppsala University, P.O. Box 591, 751 24 Uppsala, Sweden; eva.brittebo@farmbio.uu.se; 3 Department of Biochemistry, Federal University of Santa Catarina, Florianopolis 88040-970, Brazil; ariane.zamoner@ufsc.br

**Keywords:** developmental exposure, glyphosate, pesticides, melatonin, striatum, oxidative stress

## Abstract

Concerns have been raised regarding the potential adverse health effects of the ubiquitous herbicide glyphosate. Here, we investigated long-term effects of developmental exposure to a glyphosate-based herbicide (GBH) by analyzing serum melatonin levels and cellular changes in the striatum of adult male rats (90 days old). Pregnant and lactating rats were exposed to 3% GBH (0.36% glyphosate) through drinking water from gestational day 5 to postnatal day 15. The offspring showed reduced serum melatonin levels (43%) at the adult age compared with the control group. The perinatal exposure to GBH also induced long-term oxidative stress-related changes in the striatum demonstrated by increased lipid peroxidation (45%) and DNA/RNA oxidation (39%) together with increased protein levels of the antioxidant enzymes, superoxide dismutase (SOD1, 24%), glutamate–cysteine ligase (GCLC, 58%), and glutathione peroxidase 1 (GPx1, 31%). Moreover, perinatal GBH exposure significantly increased the total number of neurons (20%) and tyrosine hydroxylase (TH)-positive neurons (38%) in the adult striatum. Mechanistic in vitro studies with primary rat pinealocytes exposed to 50 µM glyphosate demonstrated a decreased melatonin secretion partially through activation of metabotropic glutamate receptor 3 (mGluR3), while higher glyphosate levels (100 or 500 µM) also reduced the pinealocyte viability. Since decreased levels of the important antioxidant and neuroprotector melatonin have been associated with an increased risk of developing neurodegenerative disorders, this demonstrates the need to consider the melatonin hormone system as a central endocrine-related target of glyphosate and other environmental contaminants.

## 1. Introduction

The extensive use of agrochemicals in food production leads to a continuous release of pesticides into the environment, making them ubiquitous pollutants that organisms and humans are exposed to [1]. The intensive use of the broad-spectrum systemic herbicide glyphosate has caused contamination of soil, surface water, and groundwater [2,3], resulting in widespread human exposure. Glyphosate has been detected both in urine samples from farmers and the general public, including children [4,5,6].

Recent epidemiological data indicate a link between glyphosate-based herbicides (GBHs) and neurological disorders in both children and adults [7,8]. Importantly, studies have shown associations between prenatal pesticide exposure and elevated prevalence of neurodevelopmental disorders [9,10]. Pesticide exposure during critical brain development periods can lead to permanent disruption in brain structures and function and, according to the Developmental Origin of Health and Disease (DOHaD) concept, increase the risk for several chronic diseases later in life [11]. Studies have demonstrated that glyphosate can induce glutamatergic excitotoxicity in the rodent brain [12] and cellular damage due to oxidative stress [13,14,15]. The association between oxidative distress, excitotoxicity, and depressive-like behavior in adult offspring previously demonstrated suggests that early-life glyphosate exposure may have a more profound impact on neurodevelopment and mental health than previously understood [15]. Oxidative stress is highly involved in neurodegenerative diseases [16,17,18], where imbalances between oxidants and antioxidant molecules can affect the onset or course of neurological disorders such as Parkinson’s disease (PD) and Alzheimer’s disease (AD) [19,20]. Exposure to glyphosate is also reported to induce significant changes in the levels of monoaminergic neurotransmitters in the striatum, prefrontal cortex, and hippocampus of rats, as well as in the expression of the neuropeptide dynorphin in the substantia nigra [21,22,23]. Taken together, this increases the concern that GBHs can negatively affect the brain.

Experimental studies further suggest that glyphosate could function as an endocrine-disrupting chemical (EDC) on the male reproductive hormonal system [24,25] and in the adrenal gland [26]. However, glyphosate-induced endocrine-related effects are mostly focused on the reproductive system, neglecting other endocrine functions such as the melatonin neuroendocrine system in the pineal gland. This tissue may be particularly susceptible to chemical exposure because it is highly perfused and not protected by the blood–brain barrier [27,28].

Melatonin is a critical hormone for circadian rhythm regulation [29]. Circadian disorders, including disruption of melatonin levels, appear early in the etiology and have been suggested as one of the primary contributing factors for the development of age-associated neurodegenerative diseases [30,31,32]. Melatonin is also a central antioxidant and free radical scavenger that functions as a neuroprotector of importance for aging and neurodegenerative diseases [33]. The pineal gland shows molecular alterations in neurodegenerative disease, which likely is related to the substantial decrease in melatonin levels observed in subjects with AD and PD [34,35]. Emerging data also indicate that melatonin acts as an epigenetic regulator during development [36].

To our knowledge, there are no previous studies on the effects of glyphosate on melatonin production. This study was conducted to investigate long-term effects of perinatal GBH exposure on melatonin levels and oxidative brain damage in the striatum of adult male rats. Primary rat pinealocyte cultures were used to study the effects of glyphosate on melatonin production and cell viability in more detail. The results show that early-life GBH exposure can cause persistent neuroendocrine deficits that may promote long-term oxidative brain damage.

## 2. Materials and Methods

### 2.1. Chemicals

The commercial GBH formulation used was Roundup^®^ G (120 g/L glyphosate, CAS No. 38641-94-0, Reg. Nr. 4036; Monsanto Crop Sciences Denmark A/S, Hellerup, Denmark). Glyphosate (CAS No. 1071-83-6), 4′,6-Diamidino-2-phenylindole dihydrochloride (DAPI), paraformaldehyde, Triton X-100, and 3-(4,5-dimethyl-2-yl)2,5-diphenyl-2H-tetrazolium bromide (MTT) were obtained from Sigma-Aldrich Co (St Louis, MO, USA). LY341495 and phorbol-12-myristate-13-acetate (PMA) were purchased from Tocris Bioscience (Bristol, UK). Bovine serum albumin and Dulbecco’s phosphate-buffered saline (PBS) were obtained from Gibco (Invitrogen, Paisley, UK). The antibodies anti-4 hydroxynonenal (ab46544), anti-NeuN (ab177487), anti-GFAP (ab4674), anti-DNA/RNA damage (ab62623), anti-Tyrosine Hydroxylase (ab6211), and the secondary antibodies donkey anti-rabbit IgG H&L Alexa Fluor^®^ 555 (ab150074) and donkey anti-mouse IgG H&L Alexa Fluor^®^ 488 (150105) were obtained from Abcam (Cambridge, UK). The antibodies anti-SOD1 (PA5-27240), anti-GCLC (PA5-44189), anti-GPx1 (PA1-18279), and the secondary antibodies donkey anti-goat IgG Alexa Fluor Plus^TM^ Plus 488 (A32814), donkey anti-sheep IgG Alexa Fluor™ 647 (A21448), goat anti-rabbit IgG Alexa Fluor^TM^ 488 (A11034), goat anti-chicken IgY Alexa Fluor^TM^ 647 (A21449), goat anti-mouse IgG Alexa Fluor^TM^ 488 (A11029), and goat anti-rabbit IgG Alexa Fluor^TM^ Plus 555 (A32727) were obtained from Thermo Fisher Scientific (Invitrogen, Rockford, IL, USA).

### 2.2. Animals

Time-mated pregnant outbred Wistar rats (Taconic, Ejby, Denmark) were housed under a 12 h dark/light cycle (lights on at 6:00 am), with free access to water and standard pellet food (R36 Labfor; Lantmännen, Kimstad, Sweden). All experimental protocols were approved by the Regional Ethical Committee (approval numbers: C191/14 and C107/16).

### 2.3. Developmental Exposure to a Glyphosate-Based Herbicide

The rat model of developmental GBH exposure has previously been described [12,23]. A commercial GBH formulation was used to expose the animals [37,38]. Briefly, single-housed pregnant rats were randomly divided into two groups: the GBH group, provided with 3% GBH (0.36% glyphosate) in drinking water, and the control group, provided with drinking water. The GBH dose was equivalent to 70 mg of glyphosate/kg/day and was selected based on our previously published study using the same animal model [23]. This dose is lower than the No Observed Adverse Effect Level (NOAEL) of glyphosate in rats defined by EPA and EFSA for developmental and maternal toxicity (1000 mg/kg/day and 300 mg/kg/day, respectively) [39,40]. Each experimental group contained four dams. The GBH treatment occurred from gestational day 5 (GD5) to postnatal day 15 (PND15). At PND21, the offspring were weaned and group-housed with same-sex littermates (3 rats/cage) and were held without further GBH treatment until PND90. Male offspring from all GBH- and drinking water-exposed litters were used for the experiments. The final number of animals used per experiment is shown in the scatter plot graph of each figure where each dot represents a different animal. Male animals were chosen to add additional evidence and make our results comparable to our recent study showing that perinatal exposure to a glyphosate-based herbicide causes dysregulation of dynorphins and an increase in neural precursor cells in the brains of PND90 male rats [23].

#### Sample Collection and Preparation

On PND90, the rats were anesthetized with isoflurane and euthanized via decapitation, alternating between the two treatment groups to avoid circadian fluctuations in melatonin levels. Trunk blood was sampled directly at the decapitation site and allowed to clot at room temperature for one hour, followed by centrifugation at 1000× *g* for 10 min. After centrifugation, the serum aliquots were collected and stored at −80 °C until analysis. The brains were immediately removed after decapitation and frozen on dry ice, as previously described [23]. Coronal cryosections (12 µm) were obtained from both hemispheres at the level of the striatum (Bregma 2.28 mm [41]) with a cryostat microtome (Thermo Scientific, Waltham, MA, USA). The brain sections were dried under a vacuum and kept at −80 °C until further use.

### 2.4. Melatonin Analysis

ELISA was used to measure melatonin levels. In addition to the serum from the PND90 rats perinatally exposed to GBH, the secretion of melatonin by rat pinealocytes in the cell culture medium was determined after treatment with glyphosate. All samples (25 µL) were measured in duplicates, as previously described [42].

### 2.5. Immunohistochemistry

Immunofluorescent staining of brain sections was conducted to study the expression of various markers in the cells. The neuronal nuclei marker, NeuN (1:500), astroglial marker, glial fibrillary acidic protein (GFAP) (1:1000), and dopaminergic neuron marker, tyrosine hydroxylase (TH) (1:500), were used as cell-type-specific markers. Products of DNA/RNA oxidation (Oxo-8-dG: 8-Oxo-7,8-dihydro-2′-deoxyguanosine for DNA and Oxo-8-G: 8-oxo-7,8-dihydroguanosine for RNA, 1:400) and lipid peroxidation (4-HNE, anti-4 hydroxynonenal, 1:50) as well as the antioxidant enzymes superoxide dismutase 1 (SOD1) (1:1000), glutamate–cysteine ligase catalytic subunit (GCLC) (1:500), and glutathione peroxidase 1 (GPx1) (1:1000) were used to evaluate oxidative stress. The immunohistochemistry was conducted, as previously described [23] with minor modifications. In brief, the frozen sections were fixated in phosphate-buffered 4% paraformaldehyde for 1 h at room temperature. Primary antibodies were mixed with blocking solution (2% bovine serum in 0.3% Triton X-PBS (PBS/t)), applied to the section, and incubated overnight at 4 °C. Alexa Fluor 488, 555, and 647 secondary antibodies (1:500, 1:1000, or 1:1500) were used for visualization (Supplementary Tables S1 and S2). The nuclear staining dye, 4′,6-diamidino-2-phenylindole, DAPI (300 nM), was added in the final rinse and used to determine the number of cells. The images of the stained striatum sections were acquired in an Olympus BX53F2 inverted microscope (Olympus, Tokyo, Japan) with a 20× objective using constant intensity settings and exposure time for all samples. The image analysis was performed using the ImageJ software. Only images showing good separation of cells and where the majority of cells were included were used for the image analysis.

### 2.6. Primary Pinealocyte Culture

Pinealocyte cultures were prepared from dissected pineal glands of 4-week-old male Wistar rats, as previously described [42]. The suspension of single cells was resuspended in DMEM/F12, plated in poly-D-lysine-coated 96-well plates (2 × 10^5^ cells/well), and incubated at 37 °C in 5% CO_2_ for 1 h before the exposures, as described below.

#### Mechanistic In Vitro Studies

Primary pinealocytes were treated for 24 h with 100 nM to 500 µM of glyphosate. The compound was dissolved in the culture medium. Cells exposed to culture medium only were used as a control group. The supernatants were then collected for analysis of melatonin content using ELISA. The cell viability was measured using the 3-(4,5-dimethyl-2-yl)2,5-diphenyl-2H-tetrazolium bromide (MTT) assay [42]. For mechanistic studies, pinealocytes were pre-incubated for 30 min with 100 nM of the protein kinase C activator PMA (phorbol-12-myristate-13-acetate) or 2 μM of the selective group II metabotropic glutamate receptor antagonist LY341495 [42], followed by 24 h co-exposure to 100 μM glyphosate. All experiments were performed using six replicates and repeated three times starting from the preparation of cell cultures from new animals.

### 2.7. Statistics

The results were presented as mean ± standard deviation (SD). The Student’s *t*-test was used for statistical analysis of the in vivo data. The in vitro data were analyzed using one-way or two-way ANOVA followed by the Tukey–Kramer test. Group differences were considered statistically significant at *p* < 0.05. The analyses were conducted with GraphPad Prism 7 software.

## 3. Results

**Figure 1:**
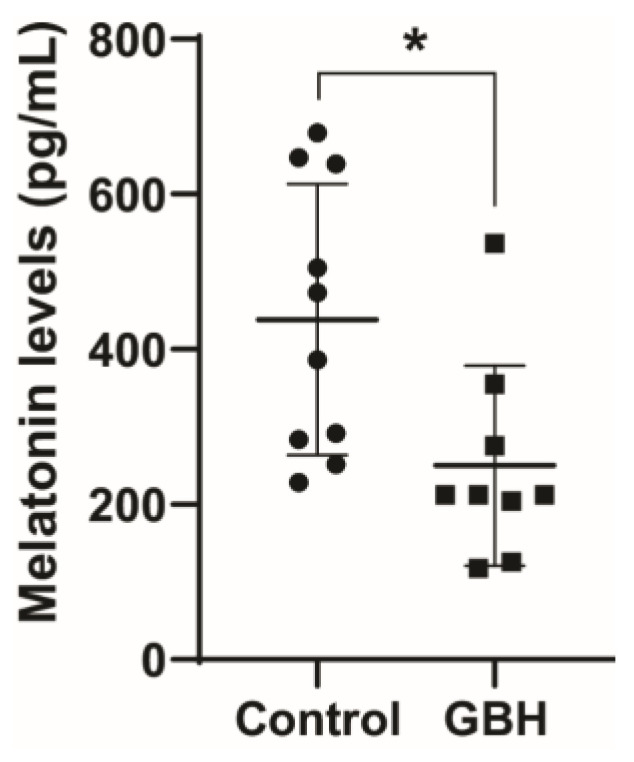
Perinatal exposure to a glyphosate-based herbicide (GBH) reduced serum melatonin levels in adult rats. Wistar rat dams were administrated 3% GBH (0.36% glyphosate) via drinking water from GD 5 up to PND15. The melatonin level was measured in serum at three months of age. Results are expressed as the mean ± SD. Statistically significant differences from the control group are indicated as follows: * *p* < 0.05 (Student’s *t*-test).

The long-term effect on serum melatonin levels was investigated by treating rat dams with GBH from GD5 to PND15 and collecting samples from the adult male offspring (PND90). The results showed that perinatal exposure to GBH significantly reduced the adult serum melatonin levels by 43% compared to the control group (Figure 1). Knowing that melatonin is neuroprotective against oxidative stress, which has been shown to play a pivotal role in GBH toxicity, we investigated markers of oxidative stress in the striatum of PND90 animals.

**Figure 2:**
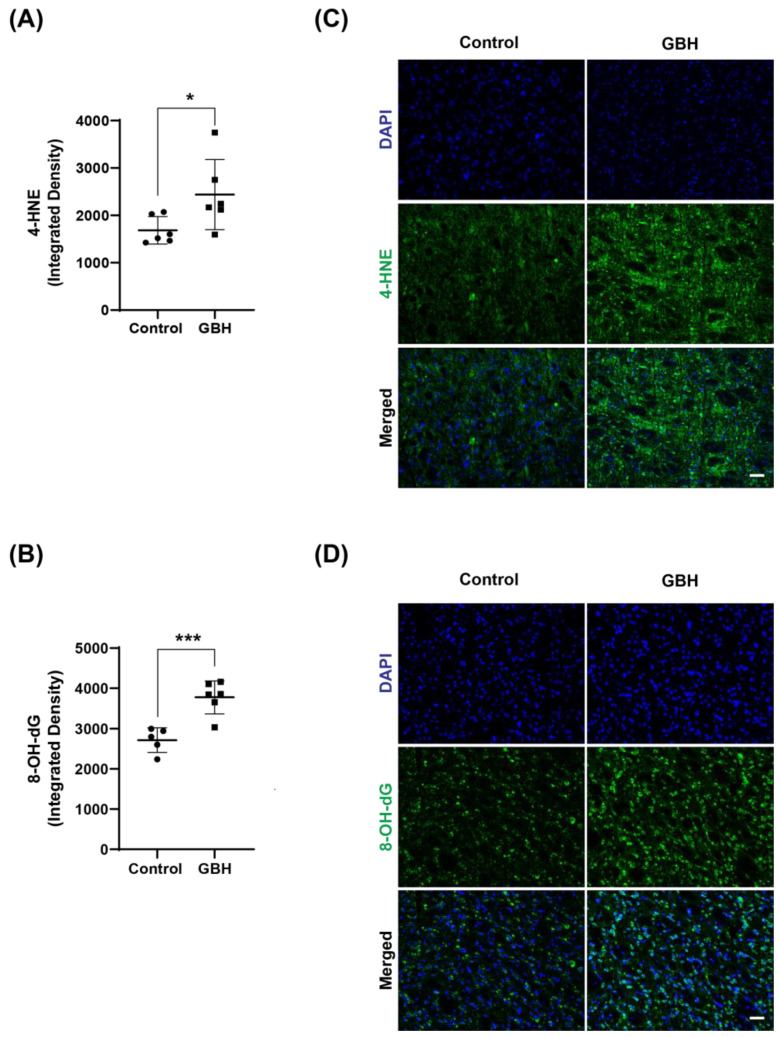
Increased oxidative stress markers in the striatum of adult rats perinatally exposed to a glyphosate-based herbicide (GBH). Wistar rat dams were administrated 3% GBH (0.36% glyphosate) via drinking water from GD 5 up to PND15. Effects of perinatal GBH exposure on lipid peroxidation (4-HNE) and DNA/RNA oxidation (8-OH-dG) were evaluated in the striatum at three months of age. The scatter plot graphs show an increase in the levels of 4-hydroxynonenal (4-HNE) (**A**) and 8-OH-dG (**B**) in the striatum of adult rats perinatally exposed to GBH. Representative fluorescent images show the presence of 4-HNE (**C**) and 8-OH-dG (**D**) in the striatum. Results are expressed as mean ± SD. Statistically significant differences from the control group are indicated as follows: * *p* < 0.05; *** *p* < 0.001 (Student’s *t*-test). Images: 20× magnification, scale bar = 50 µm.

The results revealed an increase in 4-HNE (45%, Figure 2A) and 8-OH-dG levels (39%, Figure 2B), markers of lipid peroxidation and DNA/RNA oxidation, respectively, in the GBH group compared with the control group. Representative fluorescent images of 4-HNE and 8-OH-dG are shown in Figure 2C and Figure 2D, respectively. The 8-OH-dG immunofluorescent staining was mainly localized to the cytoplasm, suggesting that mitochondrial and cytosolic nucleic acids were more affected by the GBH-induced oxidation than nuclear DNA.

**Figure 3:**
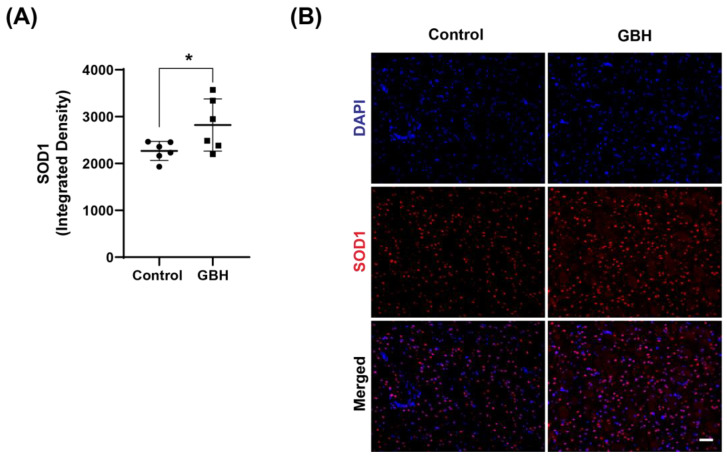
Increased levels of antioxidant enzyme superoxide dismutase 1 (SOD1) in the striatum of adult rats perinatally exposed to a glyphosate-based herbicide (GBH). Wistar rat dams were administrated 3% GBH (0.36% glyphosate) via drinking water from GD 5 up to PND15. The scatter plot graph shows an increase in SOD1 levels (**A**) in the striatum of adult rats perinatally exposed to GBH. Representative fluorescent images show the presence of SOD1 (**B**) in the striatum. Results are expressed as mean ± SD. Statistically significant differences from the control group are indicated as follows: * *p* < 0.05 (Student’s *t*-test). Images: 20× magnification, scale bar = 50 µm.

**Figure 4:**
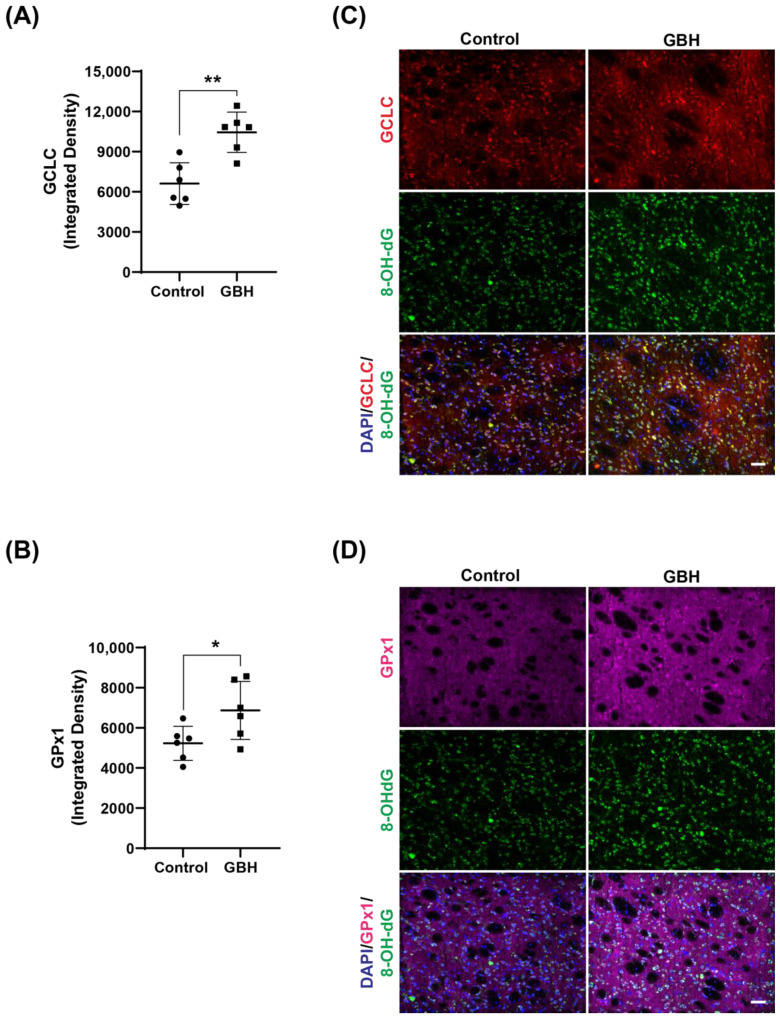
Increased levels of the antioxidant enzymes glutamate–cysteine ligase (GCLC) and glutathione peroxidase (GPx1) in the striatum of adult rats perinatally exposed to a glyphosate-based herbicide (GBH). Wistar rat dams were administrated 3% GBH (0.36% glyphosate) via drinking water from GD 5 up to PND15. The scatter plot graphs show an increase in GCLC levels (**A**) and GPx1 (**B**) in the striatum of adult rats perinatally exposed to GBH. Representative fluorescent images show the presence of the enzymes GCLC (**C**) and GPx1 (**D**), and DNA/RNA oxidation marker, 8-OH-dG (green), in the striatum. Results are expressed as mean ± SD. Statistically significant differences from the control group are indicated as follows: * *p* < 0.05; ** *p* < 0.01 (Student’s *t*-test). Images: 20× magnification, scale bar = 50 µm.

To better clarify the mechanism of oxidative stress induced by perinatal GBH exposure, we evaluated the protein levels of enzymes involved in the antioxidant defense system in the striatum of these animals at adult age. Our results showed that GBH exposure increased the levels of SOD1 (24%, Figure 3A,B), GCLC (58%, Figure 4A), and GPx1 (31%, Figure 4B). Representative fluorescent images of GCLC and GPx1 in the striatum are shown in Figure 4C,D. The merged images in Figure 4C,D show that the increase in DNA/RNA oxidation (8-OH-dG) is co-localized with increased levels of GCLC and GPx1.

**Figure 5:**
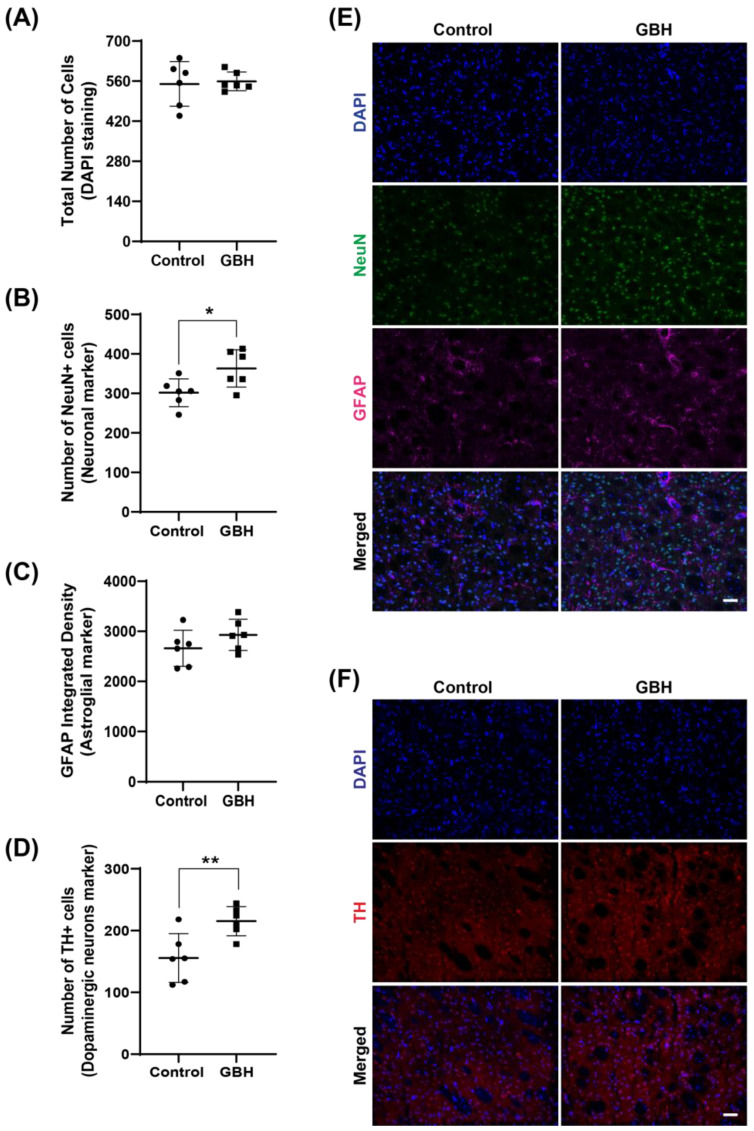
Long-term effects of perinatal exposure to glyphosate-based herbicide (GBH) on the cellular populations in the striatum of adult rats. Wistar rat dams were administrated 3% GBH (0.36% glyphosate) via drinking water from GD 5 up to PND15. Effects of perinatal GBH exposure on the total number of cells (DAPI staining, (**A**)), number of neurons (NeuN-positive cells, (**B**)), astroglial density (GFAP-integrated density, (**C**)), and dopaminergic neurons (Tyrosine hydroxylase-positive cells, (**D**)) in the striatum of adult rats (PND90). Representative fluorescent images of DAPI, NeuN, GFAP (**E**), and TH (**F**) staining in the striatum of adult rats perinatally exposed to GBH. Results are expressed as mean ± SD. Statistically significant differences from the control group are indicated as follows: * *p* < 0.05; ** *p* < 0.01 (Student’s *t*-test). Images: 20× magnification, scale bar = 50 µm.

Investigation of the cell populations in the striatum demonstrated no effects on the total number of cells (Figure 5A), while the number of neuronal cells (NeuN-positive cells) increased in the GBH group compared to the control group (20%, Figure 5B). No group differences were observed in GFAP density (Figure 5C). To further characterize the increased number of neurons, we investigated the levels of tyrosine hydroxylase (TH), a marker for dopaminergic neurons. The results showed an increase in TH-positive cells in the striatum of the GBH-treated animals (38%, Figure 5D). Representative fluorescent images of DAPI, NeuN, GFAP, and TH in the striatum are shown in Figure 5E and Figure 5F, respectively.

**Figure 6:**
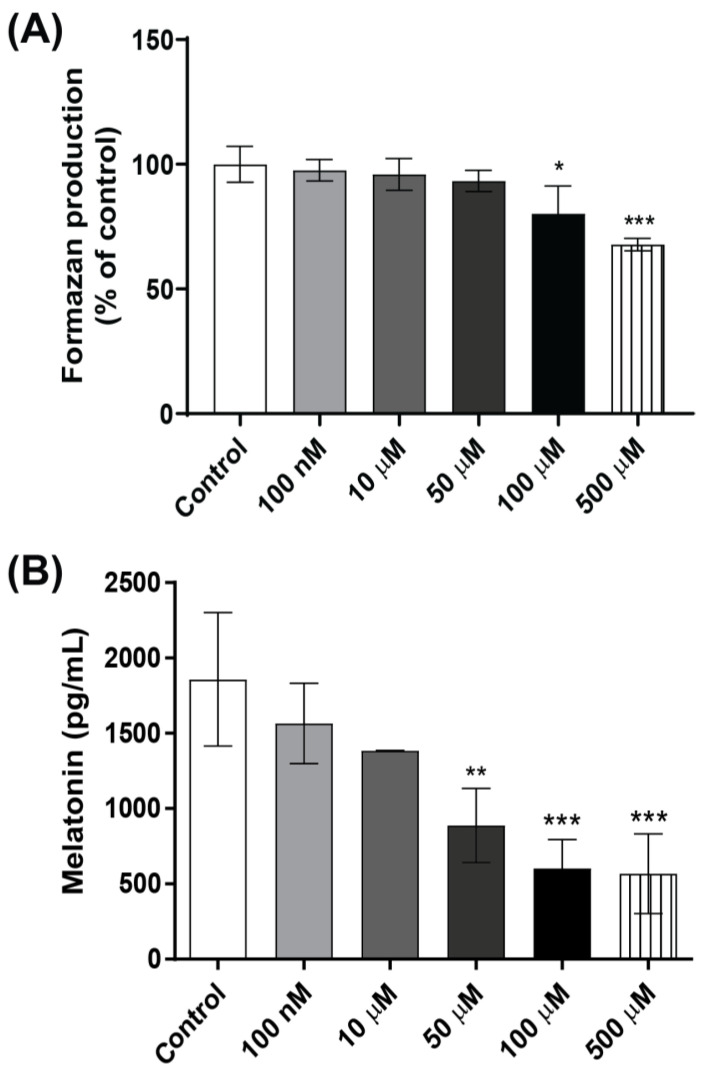
Effects of glyphosate in pinealocyte cultures. Pinealocyte viability was measured using the MTT assay (**A**) and melatonin levels in the cell medium by ELISA (**B**) after treatment with 100 nM to 500 µM glyphosate for 24 h. Results are expressed as mean ± SD of three independent experiments, based on three pinealocyte preparations (consisting of six replicates). MTT assay results are plotted as % of control. Statistically significant differences from controls are indicated as follows: * *p* < 0.05; ** *p* < 0.01 and *** *p* < 0.001 (one-way ANOVA followed by the Tukey–Kramer test).

Mechanistic in vitro studies were conducted to further investigate the effects of glyphosate exposure on pinealocytes. The MTT assay revealed that exposure to 100 µM glyphosate significantly reduced the cell viability by 20%, while 500 µM reduced the cell viability by 33% compared with controls in the primary rat pinealocyte cultures (Figure 6A). The analysis of melatonin in the cell culture medium showed a dose-dependent decrease. The lowest concentrations tested, 100 nM and 10 µM glyphosate, reduced the melatonin levels by 14% and 20%, respectively, but the differences did not reach statistical significance, while 50 µM glyphosate significantly reduced the melatonin secretion by 52% compared to the control group (Figure 6B) without affecting cell viability. Exposure to the cytotoxic concentrations (100 or 500 µM) reduced the melatonin secretion by 67% and 69%, respectively.

**Figure 7:**
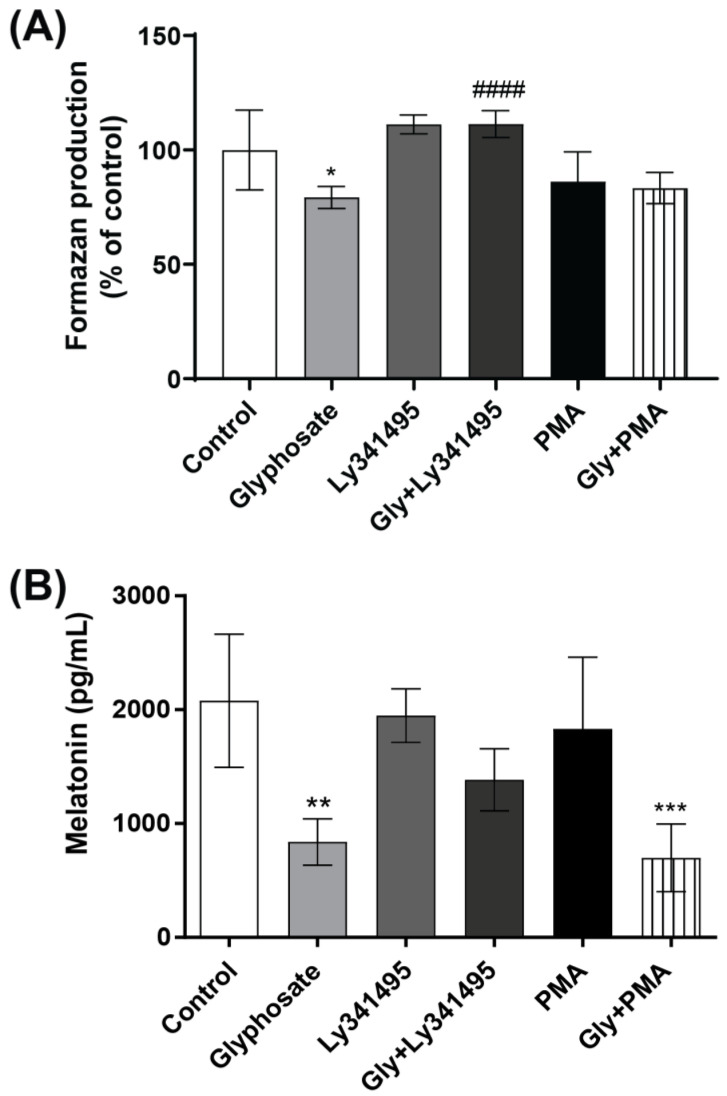
The role of PKC and metabotropic glutamate receptors in the glyphosate-induced decrease in pinealocyte viability and melatonin secretion. Pinealocytes were pre-incubated for 30 min with the mGluR3 antagonist LY341495 (2 µM) or the PKC activator PMA (100 nM), followed by co-treatment with 100 µM glyphosate for 24 h. Pinealocyte viability was measured using the MTT assay (**A**) and melatonin levels in the cell medium by ELISA (**B**). Results are expressed as mean ± SD of three independent experiments based on three pinealocyte preparations (consisting of six replicates). MTT assay results are plotted as % of control. Statistically significant differences are indicated as follows: * *p* < 0.05; ** *p* < 0.01 and *** *p* < 0.001 when compared with the control group, #### *p* < 0.0001 when compared with the glyphosate group (two-way ANOVA followed by the Tukey–Kramer test).

Since the metabotropic glutamate receptor 3 (mGluR3) and protein kinase C (PKC) have key roles in melatonin synthesis regulation [43,44], we used the group II metabotropic glutamate receptor antagonist LY341495 and the PKC activator PMA to investigate mechanisms by which glyphosate decreases pinealocyte viability and melatonin secretion. LY341495, but not PMA, prevented the reduced pinealocyte viability and partially the decrease in melatonin secretion following exposure to glyphosate. Exposure of pinealocytes to LY341495 or PMA alone did not change the cell viability or melatonin secretion (Figure 7A,B).

## 4. Discussion

The present study revealed that the melatonin hormone system is a novel endocrine-related target of glyphosate, the most used herbicide worldwide. The animal model demonstrated reduced serum melatonin levels and significantly increased striatal oxidative stress in adult rats perinatally exposed to GBH. The results demonstrated an increased DNA/RNA oxidation and lipid peroxidation as well as increased protein levels of the antioxidant enzymes SOD1, GCLC, and GPx1. This suggests that early-life exposure to glyphosate can induce long-term neuroendocrine impairments and an imbalance in the redox homeostasis. The mechanistic results also revealed that glyphosate can directly target and decrease melatonin synthesis in pinealocytes and decrease the cell viability at higher concentrations.

The decreased serum melatonin levels observed in adult rats developmentally exposed to GBH can have several important consequences. Melatonin exerts neuroprotective effects, especially against oxidative stress [45,46], and decreased melatonin levels are suggested to be involved in the development of various neurological disorders [47], including PD and AD [48]. This neuroendocrine hormone increases the resistance of neurons under enhanced oxidative/nitrosative stress, acting as a free radical scavenger and antagonist of mitochondrial radical formation as well as regulating antioxidant and pro-oxidant enzymes [49,50]. The reduced melatonin levels can therefore impair the redox homeostasis and antioxidant defense system, increasing the levels of reactive oxygen species (ROS) in the brain leading to DNA/RNA and lipid oxidation, which culminate in oxidative damage. Oxidative distress, which seems to play a pivotal role in GBH toxicity, has been related to the etiology of several chronic diseases such as neurodegenerative and neuropsychiatric disorders. In addition, toxicants that can elicit lipid peroxidation in neurons could cause neuronal dysfunction and increase the risk for neurological disorders [51].

The GBH-induced imbalance in the redox homeostasis also involved increased striatal levels of the antioxidant enzymes SOD1, GPx1, and GCLC. SOD and GPx are key enzymes in the first line of the antioxidant defense system against free radicals [52]. SOD controls superoxide anion and hydrogen peroxide generation, whereas GPx reduces hydrogen peroxide to water. Glyphosate exposure has previously been shown to modulate the expression of SOD1 by enhancing its expression in neural stem cells from the subventricular zone of postnatal mice [53]. Although the current study did not measure the activity of these antioxidant enzymes, others have demonstrated increased SOD activity in the hippocampus of young adult rats after subchronic exposure to GBH [15]. In addition, studies have reported increased GPx gene expression [54] and activity [55] in the brain of PND90 rats perinatally exposed to GBH. GCLC, also known as gamma-glutamylcysteine synthetase, is the first rate-limiting enzyme in *de novo* of glutathione biosynthesis [56]. Changes in the expression and activity of this enzyme lead to a decline in glutathione levels [57]. We have previously shown that perinatal exposure to GBH decreases the levels of glutathione in the hippocampus of rat offspring at PND15 [15]. Interestingly, GCLC is induced by the major product of lipid peroxidation, 4-hydroxynonenal (4-HNE) [57,58], which was found to be increased in the GBH-treated animals. Therefore, the increased protein levels of these important antioxidant enzymes may represent an adaptive response against the oxidative insult exerted by GBH. However, the induction of these enzymes is not fully protective as the animals have significant oxidative stress-related changes in the brain. This may indicate that the reduced melatonin level plays an important role in the oxidative distress observed in adult animals perinatally exposed to GBH.

Our results further demonstrated an increase in the number of dopaminergic neurons (TH-positive) in the striatum of adult rats following perinatal GBH exposure. TH-positive neurons are reported to be reactive to dopaminergic perturbations [59]. An increased number of dopaminergic neurons in the striatum has been shown in several studies using a parkinsonian rat model, where striatal dopamine concentrations are low [60,61,62]. PD is caused by the degeneration of dopaminergic neurons in substantia nigra that leads to a reduction in striatal dopamine levels. Studies suggest that the adult striatum may retain some intrinsic ability to exert a compensatory proliferation of TH-positive neurons to act as a local source of dopamine in response to the progressive destruction of the nigrostriatal pathway [63,64,65]. The increase in TH-positive neurons in the adult striatum reported here could, therefore, be a response to a dopaminergic insult in the substantia nigra. In line with this, we have previously demonstrated that the levels of the neuropeptide dynorphin, known to also act as a protector of dopaminergic neurons [66,67], are decreased in the substantia nigra of adult offspring following perinatal GBH exposure [23]. In addition, acute exposure to glyphosate decreased basal extracellular dopamine levels in the striatum of young adult male rats [22], and chronic exposure to GBH caused a decrease in the number of dopaminergic neurons in the substantia nigra pars compacta of mice [68]. However, whether the increase in TH-positive cells in the striatum of adult rats perinatally exposed to GBH is a compensatory mechanism for a loss of dopaminergic neurons in the substantia nigra, or due to some other mechanisms, needs to be further investigated.

The mechanistic in vitro studies demonstrated that glyphosate induced a concentration-dependent effect on pinealocytes. Pinealocytes produce melatonin and negatively regulate its synthesis by secreting glutamate via an exocytic mechanism. Glutamate binds to mGluR3 on the pinealocyte membrane and partially blocks melatonin synthesis through an inhibitory cAMP cascade and inhibition of serotonin N-acetyltransferase [43,69]. The glyphosate exposure directly inhibited the melatonin synthesis at lower concentrations, partially through group II metabotropic glutamate receptors, and affected both the cell viability and melatonin secretion at the higher concentrations, which still are lower than the glyphosate levels found in human blood and urine samples after acute intoxication [70]. This suggests that the perinatal GBH exposure caused the effects in the melatonin endocrine system by inducing pineal gland dysfunction and reduction in melatonin levels through glutamate-related mechanisms. Developmental exposure to a GBH formulation has previously been reported to trigger oxidative stress and glutamate excitotoxicity through glutamate receptor activation and voltage-dependent calcium channels opening in immature offspring hippocampus [12]. Melatonin has also been shown to have an important role in maintaining calcium homeostasis by preventing glutamate-induced increases in intracellular calcium levels [71,72]. In addition, molecular dynamic simulation has demonstrated that due to the structural similarity with glutamate, glyphosate may be able to bind to glutamate receptors resulting in an over-activation of these receptors [15]. Interestingly, the environmental toxin β-N-methylamino-L-alanine (BMAA), which also acts as a glutamate receptor agonist, was recently shown to target the pineal gland and decrease melatonin levels in adult rats following a short postnatal exposure as well as in primary pinealocytes through glutamatergic mechanisms [42]. This indicates that the pineal gland and the melatonin hormone system could be a vital, but currently neglected, primary target for environmental pollutants and particularly those that act on the glutamatergic system.

Oral exposure to a commercial GBH formulation was used in the present animal model to better represent occupational exposure to this pesticide. Therefore, it remains to be elucidated if the observed long-term effects in PND90 rats are due to glyphosate alone or the synergism between this herbicide and inert substances present in its commercial formulation. There is evidence showing glyphosate permeability across the blood–brain barrier, potentially via amino acid transporters [73,74], and glyphosate has beendetected in the brain [75] and cerebrospinal fluid [76] after human exposure. Even though several of the co-formulants present in GBH remain confidential information, no studies have shown the capacity of the main known surfactants used in the European Union prior to 2016, i.e., the polyoxyethylene tallow amine (POEA) class, to cross the blood–brain barrier. Our mechanistic in vitro studies of glyphosate-treated pinealocyte cells also show that the decrease in melatonin levels is triggered by glyphosates via activation of mGluR3, therefore, indicating that the effect on the melatonin endocrine system after in vivo exposure to GBH is likely due to glyphosate itself.

## 5. Conclusions

This study revealed reduced serum levels of the key antioxidant melatonin and oxidative stress-related changes in the striatum of adult rats perinatally exposed to the commercial formulation of glyphosate. The GBH exposure also increased the number of dopaminergic neurons in the striatum. Mechanistic in vitro studies in primary pinealocytes demonstrated that glyphosate reduced cell viability at the higher concentrations used (100 or 500 µM), while 50 µM glyphosate directly inhibited melatonin synthesis through partial activation of mGluR3. Considering that melatonin is an important neuroprotector in the brain and decreased melatonin levels have been associated with neurodegenerative disorders, this illustrates the need to start to consider the melatonin hormone system as a primary target for glyphosate and other environmental contaminants.

## Data Availability

The data presented in this study are available on request.

## Supplementary Materials

The following supporting information can be downloaded at: https://www.mdpi.com/article/10.3390/antiox12101825/s1, Table S1: List of primary antibodies used for immunofluorescence analysis.; Table S2: List of secondary antibodies used for immunofluorescence analysis.
