# Supplementary material for: Long-Term Effects of Perinatal Exposure to a Glyphosate-Based Herbicide on Melatonin Levels and Oxidative Brain Damage in Adult Male Rats
Source: Antioxidants (Basel). 2023 Oct 3;12(10):1825. doi: 10.3390/antiox12101825 (PMC10604376; doi:10.3390/antiox12101825)
Supplement: Supplementary file 1 [file antioxidants-12-01825-s001.zip › antioxidants-2599850-supplementary.pdf]

Table S1. List of primary antibodies used for immunofluorescence analysis.

Antibody	Brand	Cat. number	Dilution	Description
4HNE	Abcam	Ab46544	1:50	Goat polyclonal to 4 Hydroxynonenal
8-OH-dG	Abcam	Ab62623	1:400	Mouse monoclonal (15A3) to DNA/RNA damage
SOD1	Invitrogen	PA5-27240	1:1000	Rabbit polyclonal to SOD1
GCLC	Invitrogen	PA5-44189	1:500	Mouse polyclonal to GCLC
GPx1	Invitrogen	PA1-18279	1:1000	Sheet polyclonal antibody to GPX1
NeuN	Abcam	Ab177487	1:500	Rabbit monoclonal (EPR12763) to NeuN – Neuronal Marker
GFAP	Abcam	Ab4674	1:1000	Chicken polyclonal to GFAP
TH	Abcam	Ab6211	1:500	Rabbit polyclonal to Tyrosine Hydroxylase

Table S2. List of secondary antibodies used for immunofluorescence analysis.

Secondary antibody	Brand	Cat. Number	Dilution	Fluorescence	Primary Ab
Donkey anti-Goat IgG	Invitrogen	A32814	1:1000	Alexa Fluor™ Plus 488	HNE
Goat anti-mouse IgG	Invitrogen	A11029	1:500	Alexa Fluor™ 488	8-OH-dG
Donkey anti-mouse IgG	Abcam	Ab150105	1:500	Alexa Fluor® 488	8-OH-dG
Donkey anti-rabbit IgG	Abcam	Ab150074	1:1000	Alexa Fluor® 555	GCLC
Donkey anti-sheep IgG	Invitrogen	A21448	1:500	Alexa Fluor™ 647	GPx1
Goat anti-rabbit IgG	Invitrogen	A11034	1:500	Alexa Fluor™ 488	NeuN
Goat anti-chicken IgY	Invitrogen	A21449	1:1500	Alexa Fluor™ 647	GFAP
Goat anti-rabbit IgG	Invitrogen	A32732	1:500	Alexa Fluor™ Plus 555	TH and SOD1
